# Plasma Albumin Changes During Admission for Bloodstream Infection as Prognostic Predictors After Discharge—A Population-Based Cohort Study

**DOI:** 10.3390/jcm15114329

**Published:** 2026-06-03

**Authors:** Kim Oren Gradel, Ram Benny Dessau, Jens Kjølseth Møller, Stig Lønberg Nielsen, John Eugenio Coia, Pedro Póvoa

**Affiliations:** 1 Center for Clinical Epidemiology, Odense University Hospital, 5000 Odense, Denmark; pedrorpovoa@gmail.com; 2 Research Unit of Clinical Epidemiology, Department of Clinical Research, University of Southern Denmark, 5230 Odense, Denmark; 3 Department of Clinical Microbiology, Sjællands Universitets Hospital, 4000 Slagelse, Denmark; ramd@regionsjaelland.dk; 4 Institute for Regional Health Research, University of Southern Denmark, 5230 Odense, Denmark; jens.kjoelseth.moeller@rsyd.dk (J.K.M.); jcoia@health.sdu.dk (J.E.C.); 5 Department of Clinical Microbiology, Vejle Hospital, University Hospital of Southern Denmark, 7100 Vejle, Denmark; 6 Department of Infectious Diseases, Odense University Hospital, 5000 Odense, Denmark; stig.nielsen@rsyd.dk; 7 Research Unit of Infectious Diseases, Department of Clinical Research, University of Southern Denmark, 5230 Odense, Denmark; 8 Department of Intensive Care, Hospital de São Francisco Xavier, Unidade Local de Saúde de Lisboa Ocidental, Estrada do Forte do Alto do Duque, 1449-028 Lisbon, Portugal; 9 NOVA Medical School, Nova University of Lisbon, 1600-560 Lisbon, Portugal; 10 D’Or Institute for Research and Education (IDOR), Rio de Janeiro 22281-100, Brazil

**Keywords:** bloodstream infections, plasma albumin, prognosis, discharged alive, short-term mortality, long-term mortality

## Abstract

**Background:** With a half-life of 18–20 days, rapid declines in plasma albumin (PA) levels may reflect increased vascular loss of PA, e.g., as seen with inflammatory insults. **Methods:** Our study included 11,562 adult patients with first-time bloodstream infection (BSI) in a geographically well-defined Danish region between 2007 and 2016, all with ≥2 PA specimens during BSI admission and discharged alive from the hospital. We assessed mortality 1–30, 31–90, 91–365, and >365 days after discharge. Predictors were the BSI admission’s first or last PA specimen’s level, grouped into <25, 25–34, and ≥35 g/L as well as combinations of these (reflecting changes [none, increase, or decrease]). We applied Cox’s regression analyses for a baseline model with age, sex, comorbidity, and BSI microorganisms as well as the baseline model with amendments of the first, last, or change in the PA levels. We further computed areas under the ROC curves (AUROCs) to assess how much the PA covariates changed AUROCs for the baseline model. **Results:** The last PA group and the changes between the first and the last PA group were the strongest predictors, with little differences between these. Lower PA level groups predicted higher mortality, especially up to 90 days. For 1–30 day mortality, the hazard ratio was 3.69 for the last PA group of <25 g/L and 0.31 for ≥35 g/L (reference: 25–34 g/L). AUROCs for the baseline model were 0.72 for the 1–30 and 0.73 for the 31–90-day mortality whereas the amendment of the PA changes increased these areas to 0.79 and 0.76, respectively. Higher AUROCs (range 0.83–0.90) were seen in non-comorbid patients, in patients aged <65 years, and in the lower quartile of days between the first and the last PA specimen. **Conclusions:** The BSI admission’s last PA specimen was a strong mortality predictor, especially up to 90 days. Higher AUROCs were found in younger, non-comorbid patients, and in patients with higher velocity of the PA changes. These results corroborate that hypoalbuminemia is mainly a marker of acute events.

## 1. Introduction

Hypoalbuminemia is well-known as a strong predictor of adverse outcomes in many different patient conditions [1,2,3]. Causes of hypoalbuminemia comprise reduced synthesis, increased catabolism, extravasation, or external loss [4], with more than one mechanism often involved in the individual patient. As the half-life of plasma albumin (PA) is 18–20 days [5,6], changes in PA levels over shorter time periods (hours or a few days) mainly reflect an acute condition, such as that encountered after surgery or a bloodstream infection (BSI) [4,7]. As the PA level is often inversely correlated with the degree of inflammation, e.g., assessed by the C-reactive protein (CRP) level [8,9], it is classified as a negative acute phase protein [10].

Most prognostic studies on PA have included one-time measures whereas fewer studies have assessed the prognostic impact of changes in PA. Moreover, most studies only report in-hospital mortality as post-discharge follow-up is often difficult. One study of 3967 patients with sepsis evaluated both PA at admission and one week later as predictors of in-hospital mortality [11]. To our knowledge, only one study has incorporated both changes in PA levels during a hospital admission and the prognosis after discharge [12].

Most of the patients with a BSI are discharged alive from the hospital. However, as these patients are often old and comorbid [13,14], their prognosis remains unfavorable after discharge, e.g., as seen in the few studies of the 1-year mortality approaching 50% [15,16]. A BSI is thus often a frailty marker of a severe underlying condition, e.g., cancer or a weakened immune system.

We have a population-based cohort of patients with BSI [14] in which 11,562 patients were discharged alive and had two or more PA specimens sampled during the admission with their first BSI episode. We aim to evaluate the discriminatory ability of changes between the BSI admission’s first and last PA specimen in the prediction of mortality 1–30, 31–90, 91–365, and >365 days after discharge, in comparison to one-time levels of either the first or the last PA specimen.

## 2. Materials and Methods

### 2.1. Setting

The Danish healthcare system, administered by five geographically well-defined regions, is tax-funded and thus without charge for individual patients [17]. As the private hospital sector mainly performs elective surgery, virtually all acutely ill patients are admitted to a public hospital in the region where they reside. All Danish residents have a unique Danish personal identifier used in all health and administrative registries, which enables linkage between these [18].

### 2.2. Study Population

The study population has been described previously [14]. It includes all patients with one or more BSIs from 2007 through 2016 in two Danish regions, but we only had biochemistry data from the Region of Southern Denmark (RSD). The RSD has about 1,260,000 residents, equivalent to 21% of the Danish population of 6 million.

**Figure 1:**
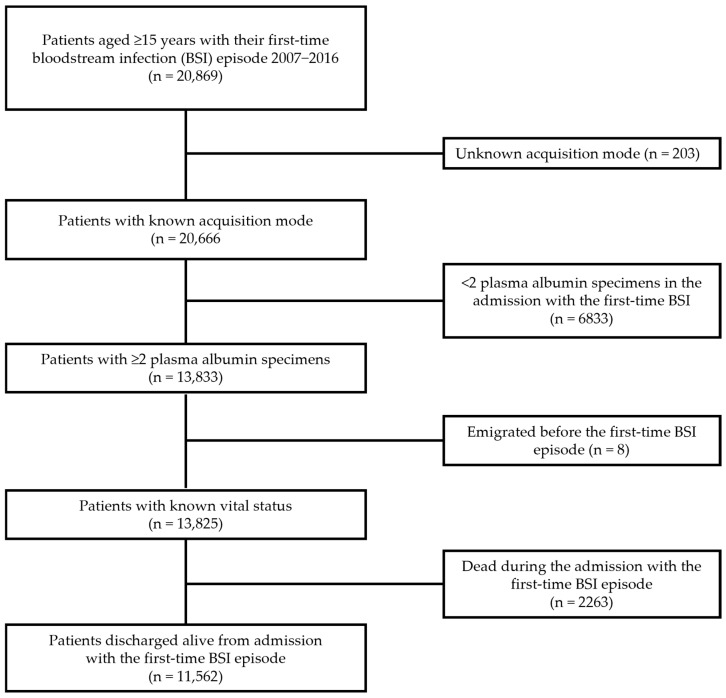
Flowchart of the study population.

In brief, we initially retrieved data for all positive blood cultures (BCs) from the laboratory system MADS [19], using globally accepted criteria to compute BSI episodes [20,21]. We included patients who were 15 years or older when encountering their first BSI episode (Figure 1).

The BC data were linked to the Danish National Patient Registry, with recordings of diagnoses and procedures for all inpatients since 1977 [22]. We computed all inpatient admissions with an admission and a discharge date for each admission. For 99.0% of the patients, the date of sampling the first positive BC that resulted in their first BSI episode (the BSI date) fell within an admission and a discharge date, which enabled the computation of the acquisition mode (Figure 1). We computed the acquisition mode as either community-acquired (CA), healthcare-associated (HCA), or hospital-acquired (HA) using generally accepted algorithms [20,23]. We excluded patients with <2 PA specimens during the admission with the first BSI-episode (hereafter designated the BSI admission), who had emigrated before their first BSI episode and who died during the BSI admission (Figure 1). For the final study population, we computed the Charlson comorbidity index (CCI) [24] from discharge diagnoses recorded in the Danish National Patient Registry prior to or during the BSI admission.

### 2.3. Statistical Analyses

We divided the BSI admission’s first and the last albumin specimen’s level into 0–24, 25–34, and ≥35 g/L, representing severe hypoalbuminemia, hypoalbuminemia, and normal PA levels, respectively. We combined these categories into nine first/last specimen groups, thus rendering patient groups of declining change (25–34/0–24, ≥35/0–24, or ≥35/25–34 g/L), no change (0–24/0–24, 25–34/25–34, or ≥35/≥35 g/L), or increasing change between the categories (0–24/25–34, 0–24/≥35, or 25–34/≥35 g/L). We further commented on whether PA levels declined, were unchanged, or increased between the first and the last specimen, including for the 0–24/0–24, 25–34/25–34, and ≥35/≥35 g/L categories.

For these nine patient groups, we computed contingency tables with baseline patient characteristics (age, sex, acquisition mode [CA, HCA, HA], the CCI [0, 1–2, and ≥3 points], and main groups of microorganisms in the first BSI episode [mono-microbial Gram-negatives, mono-microbial Gram-positives, mono-microbial fungi, and poly-microbial]).

We depicted Kaplan–Meier mortality curves for the nine patient groups, with mortality as the outcome from the day after until 365 days after discharge from the BSI admission.

We determined death 1–30, 31–90, 91–365, and >365 days after discharge from the BSI admission as our four outcomes. We commented on trends in mortality within each of these periods in relation to the first, last, and first/last categories. For each outcome, we applied Cox’s regression analyses with hazard ratios (HRs) and 95% confidence intervals (CIs). Initially, we applied a baseline model including age, sex, acquisition mode, CCI group, and group of microorganisms (*Escherichia coli*, *Enterobacter* spp., *Klebsiella* spp., other Enterobacteriaceae, *Pseudomonas aeruginosa*, anaerobic Gram-negative rods, other Gram-negatives, *Staphylococcus aureus*, coagulase-negative staphylococci, *Streptococcus pneumoniae*, hemolytic streptococci, enterococci, other Gram-positive cocci, Gram-positive rods, fungi, poly-microbial). We then added either the nine patient groups (with the 25–34/25–34 g/L group as the reference), the first PA category (with the 25–34 g/L group as the reference), or the last PA category (with the 25–34 g/L group as the reference) to the baseline model. For each of the four outcomes, follow-up ended with death, 31 December 2019 (date of the last recording of the vital status), emigration, and, for the day 1–30, 31–90, and 91–365 mortality, day 30, 90, and 365, respectively, whichever came first. For all the Cox’s regression models, we computed the area under the receiver operating characteristic curve (AUROC) and compared these mutually [25,26]. All analyses were reiterated in the following subgroups: age groups (15–64, 65–79, and ≥80 years), CCI groups (0, 1–2, and ≥3 points), quartiles of days between the first and the last PA specimen, and patients with liver or kidney diseases registered in the Danish National Patient Registry prior to or during the BSI admission.

We used Stata^®^, vs. 18, (StataCorp., College Station, TX, USA) for the analyses. Significance was defined as 95% CIs not overlapping 1 or a two-sided *p*-value of <0.05.

## 3. Results

### 3.1. Baseline Characteristics

The final study population comprised 11,562 patients with their first BSI episode in the RSD between 2007 and 2016 (Figure 1).

The patients were typically old (median age 72 years) and 77.6% had recorded comorbidity (Table 1). More than half of the BSI episodes (53.2%) were CA, 29.1% were HCA, and 17.7% were HA. As regards main microorganism groups, 52.3% of the BSI episodes had mono-microbial Gram-negatives, 39.8% had mono-microbial Gram-positives, 1.6% had mono-microbial fungi, and the remaining 6.4% were poly-microbial.

Among the 656 patients (5.7%) with a PA level of <25 g/L in their first specimen, a trend of younger patients, more females, less HCA, more HA, and fewer CCI points were seen in parallel in the groups going from <25/<25, over <25/25–34, to <25/≥35 g/L (Table 1). The distribution between Gram-negative organisms and Gram-positive organisms changed considerably between <25/25–34 and <25/≥35 g/L (Gram-negative organisms 50.3% vs. 29.5%, Gram-positive organisms 37.2% vs. 52.5%, respectively).

For the 4851 patients (42.0%) with a PA level between 25 and 34 g/L in their first specimen, the trends as reported for patients with <25 g/L in their first PA specimens were less consistent, with CCI as an exception (Table 1).

Among the 6055 patients (52.4%) having a PA level of ≥35 g/L in their first specimen, a trend of lower age, more CA, more HCA, less HA, and fewer CCI points were seen parallel to groups going from ≥35/<25, over ≥35/25–34, to ≥35/≥35 g/L (Table 1).

### 3.2. Overall Fluctuations Between the First and the Last PA Levels

Groupwise, 3445 (29.8%) declined (i.e., the 25–34/0–24, ≥35/0–24, and ≥35/25–34 g/L groups), 6831 (59.1%) were unchanged (i.e., the <25/<25, 25–34/25–34, and ≥35/≥35 g/L groups), and 1286 (11.1%) increased (i.e., the 0–24/25–34, 0–24/≥35, and 25–34/≥35 g/L groups) between the first and the last PA specimens (Table 1). When looking at the 6831 patients with an unchanged group, 3979 (58.3%) declined, 804 (11.8%) were unchanged, and 2048 (30.0%) increased from the first to the last PA specimen (Table A1). Thus, for the whole study population of 11,265 patients, 7424 (64.2%) declined, 804 (7.0%) were unchanged, and 3334 (28.8%) increased their PA level from the first to the last specimen.

### 3.3. Mortality in the Patient Groups

**Figure 2:**
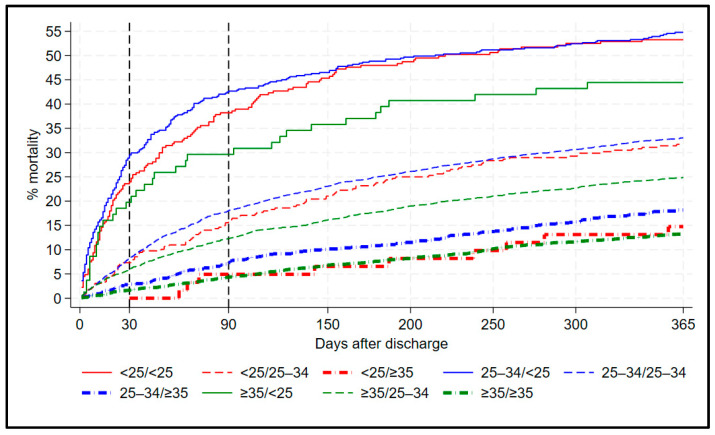
Kaplan–Meier mortality curves for nine patient groups having combinations of <25, 25–34, or ≥35 g/L plasma albumin in their first or last specimen, e.g., “<25/25–34” have <25 g/L in their first and 25–34 g/L in their last specimen. The two dashed lines denote 30 and 90 days after discharge.

The nine Kaplan–Meier curves could be divided into three groups (Figure 2).

The group with the three curves with the highest mortality had <25 g/L in the last PA specimen, an intermediate group had 25–34 g/L in the last PA specimen, and the group with the lowest mortality had ≥35 g/L in the last PA specimen. Within each of these three groups, patients with a first PA level of 25–34 g/L had the highest mortality, followed consecutively by patients with <25 g/L and ≥35 g/L, but with diminishing differences from <25 g/L to ≥35 g/L in the last specimen.

For all mortality time periods and within both the first and the last level group, patients with a PA level of <25 g/L had higher mortality than the 25–34 g/L group who had higher mortality than the ≥35 g/L group (Table 2). When comparing the first and the last level groups, the main differences in mortality were found for <25 g/L with the last level group having higher mortalities in all time periods. Within each of the three first-level groups, lower mortality in all time periods was observed in parallel with the last PA level of <25 g/L, over 25–34 g/L, to ≥35 g/L.

### 3.4. Adjusted Cox’s Regression Analyses

A total of 29 patients (0.25%) were censored prior to the end, all due to emigration, with three, four, 10, and 12 emigrating in the 1–30, 31–90, 91–365, and ≥365-day period, respectively. The study population of 11,562 patients had a total follow-up time of 43,063 years and a median follow-up time of 42 months for each patient.

As regards changes between the first and the last specimen, the highest HRs for the 1–30-day mortality (ranging from 2.54 to 3.67) were seen in the three groups having the last PA level of <25 g/L (Table 3). The lowest HRs were seen in two groups of ≥35 g/L in the last specimen, 0.40 in the 25–34/≥35 group, and 0.25 in the ≥35/≥35 group.

In the first two periods (1–30 and 31–90 days), HRs for the last level of <25 g/L were higher than HRs for the first level of <25 g/L (3.69 vs. 1.59 and 2.02 vs. 1.36, respectively) (Table 3). For the first vs. the last levels of ≥35 g/L, HRs were <1 in the same periods and they were lowest for the last level.

In all analyses, HRs approached 1 with increasing mortality length period.

### 3.5. Discriminatory Ability Based on the Adjusted Cox’s Regression Analyses

The AUROC of the baseline model (comprising sex, age, acquisition, comorbidity, and microorganisms) differed little between the four time periods (1–30, 31–90, 91–365, and >365-day mortality) ranging from 0.72 to 0.73 (Table A2). The first and last PA group increased the AUROC to 0.74 and 0.78, respectively, for the 1–30-day period and to 0.75 and 0.76 for the 31–90-day period whereas the increase was smaller beyond day 90. The increases in AUROCs of the models with PA changes from the first to the last PA group were minor compared to the last PA group models.

### 3.6. Analyses in Subgroups of Age, CCI, and Quartiles of Time Between the First and the Last PA Specimen

In all subgroups, the adjusted HRs did not differ materially from the overall ones reported in Table 3 although many 95% CIs were wide. Likewise, within each subgroup, all AUROCs showed the same increasing trends as reported in Table A2, with minor differences between the last PA specimen and the change between the first and the last PA specimen. Hence, we only show AUROCs for the subgroups, based on the baseline model and the baseline model with the amendment of changes between the first and the last PA specimen (Table A3). Within each mortality period, AUROCs were the highest for the youngest patients, those with 0 CCI points, and the lowest quartile and they decreased as the patients became older, more comorbid, or with higher quartiles. Likewise, AUROCs generally decreased with longer mortality periods, especially beyond day 90. Changes between the first and the last PA specimen contributed the most to the AUROC of the baseline model as regards 1–30 and 31–90-day mortality, with smaller increments thereafter.

### 3.7. Analyses in Patients with Liver or Kidney Diseases

A total of 555 and 1452 patients had liver and kidney diseases, respectively, registered as diagnoses in the Danish National Patient Registry before or during the BSI admission. Their AUROCs did not differ materially from the overall AUROCs, in particular when considering the wide CIs for the liver or kidney patients (Table A3 vs. Table A2).

## 4. Discussion

In this study with a high number of patients discharged alive after a BSI admission, lower PA levels predicted a higher mortality, especially up to around 90 days after discharge. For both the change between the BSI admission’s first and last PA specimen and its last PA specimen, the AUROC approached 0.8, which is an acceptable discriminatory ability [27]. Interestingly, AUROCs were especially high in younger patients (range 0.81–0.85 up to one year after discharge) and in patients without recorded Charlson comor-bidity (range 0.79–0.90 for all mortality periods). Shorter time spans between the first and the last PA specimen were also associated with higher AUROCs, exceeding 0.8 up to day 90. We believe that especially these major changes in AUROCs, as seen in younger and non-comorbid patients and in shorter time periods between the first and the last PA specimen, are clinically useful as they pay attention to patient groups in whom preventive measures during and after discharge may be beneficial.

Hypoalbuminemia as a predictor of a worse prognosis has been reported in numerous studies [1,28,29]. With a half-life of 18–20 days [5,6], the velocity of PA level changes indicates whether a resulting hypoalbuminemia is mainly a marker of an acute or a more chronic condition. In the present study, higher AUROCs were associated with shorter time spans between the first and the last specimens, especially for the short-term mortality. This indicates a better discriminatory ability related to quicker changes in the PA level, indicating its importance as a marker of an acute event. However, as hypoalbuminemia was also a predictor of long-term mortality, it is probably also a marker of more chronic conditions. This is hardly surprising given the many possible causes of hypoalbuminemia, which may occur concomitantly in the same patients [4,30]. A BSI is an acute event characterized by high levels of inflammation. Studies in patients with kidney disorders, mainly conducted by one study group, have reported high inverse correlations between levels of the inflammatory marker CRP and PA [31] and we found the same in patients with CA BSI [32]. The results of the present study corroborate these findings and underline PA’s role as a negative acute phase protein and consequently hypoalbuminemia as a marker of the inflammatory response [10].

We were inspired by two studies that assessed changes in PA levels as predictors of mortality [11,12]. A study of 3967 patients with sepsis admitted to an intensive care unit assessed PA levels at admission and one week later as predictors of in-hospital mortality [11]. Only the one-week PA level predicted in-hospital mortality. Interestingly, odds ratios for each 1 g/dL increment of the PA level were 0.67 for patients aged >65 years, 0.60 for 45–65 years, and 0.44 for <45 years. Moreover, AUROCs increased with the younger age groups (0.74, 0.77, and 0.83 for >65, 45–65, and <45 years, respectively). The increasing AUROCs for younger age groups accord with our results, with the difference that our outcome was post-hospital mortality. The study suggested that the PA level was more a marker of chronic conditions in the elderly, but reasons for these gradients are merely speculative and other study types than retrospective observational studies are needed. Another study comprising 276 patients admitted to a medical ward with different acute disorders assessed short- and long-term mortality with a median follow-up period of 23 months [12]. PA was measured at admission and discharge, each categorized into <34 g/L and ≥34 g/L. Hypoalbuminemia, both at admission and discharge, predicted higher mortality, with the highest mortality in patients having <34 g/L in both. In contrast to our study, the outcome included both in- and post-hospital mortality. To our knowledge, this is the only other study that has evaluated changes in PA levels and mortality beyond in-hospital mortality. As regards BSI, a few studies have assessed long-term mortality [15,16,33,34], one of which assessed 0–30 and 31–365-day mortality with a one-time level of PA as a prognostic predictor in patients with a CA BSI [34].

Of note, we found that the mean PA level was lower in the last specimen than in the first. This was also reported in the two other studies, which assessed PA changes during admission [11,12] as well as in other studies [30,35]. This is not surprising given the quick decline in the PA level in relation to a serious infection, such as a BSI, followed by a slow increase thereafter, which may take days or weeks [6,32,35]. Moreover, patients with BSI who are discharged alive are still old and comorbid and the high rates of recurrent BSI episodes [33,36], readmissions (many with an infectious disease diagnosis) [37], and long-term mortality [15,16,33,34] are all markers of frail patients.

As hypoalbuminemia may also be caused by liver or kidney diseases, we reiterated our analyses for these patients to assess whether it would impact our results. The results were materially the same as for the whole study population. Inflammation is often an important component in both liver and kidney diseases [31,38,39], which makes it even more difficult to distinguish between specific mechanisms that may cause hypoalbuminemia.

Our study has several strengths. It is population-based, includes a high number of patients with BSI, which is unambiguously defined [20,21], and it comprises many hospitals and wards. In Denmark, patients with a diagnosed BSI are always admitted to hospital, enabling the retrieval of data for, e.g., comorbidity and acquisition of the BSI from the Danish National Patient Registry [22]. Very few patients (0.25%) were censored and long-term follow-up was enabled due to the valid Danish Civil Registration System [18].

There are also several limitations which deserve further consideration. Firstly, confounding by indication is inevitable when dealing with real-life data. Our patients differed from patients discharged alive with one or no PA specimens retrieved during their BSI admission because clinicians found fewer indications for retrieving PA specimens. Table A4 shows characteristics for the 6060 patents who were discharged alive after the retrieval of 0 or 1 PA specimens. These patients were younger and had lower mortality up to day 365 whereas the other characteristics (sex distribution, acquisition of the BSI, comorbidity, and main microorganism groups) were more similar to our study cohort. Our study population also differed from patients who died during the BSI admission, but we have not elaborated further on these differences as the main aim was the assessment of post-discharge mortality. Secondly, we lacked clinical data, e.g., sepsis criteria, which could have refined our prognostic models. However, in an earlier study of 1844 adult patients with CA BSI, a one-time PA level on the BSI date was more discriminatory than the Systemic Inflammatory Response Syndrome (SIRS) criteria in the prediction of 0–30 and 31–365-day mortality [34]. Thirdly, hypoalbuminemia may partly be due to the infusion of intravenous fluids although an earlier study in patients with a CA BSI, which included trajectories for both PA and hemoglobin, indicated that this was less important [32]. Another bias could be due to albumin or plasma as components in the fluid, but these are rarely used in Danish hospitals [32]. Fourthly, the time span between the BSI and discharge date differed among the patients. We chose the discharge date as the baseline to underline that our study population only included patients discharged alive. Fifthly, the retrieval of the first and last PA specimens could differ in relation to the admission and discharge date, respectively, as this retrieval is based on clinical judgment. In patients with a CA- or HCA-BSI, 98.3% of the first PA specimens were retrieved within two days after admission, whereas this was the case for 85.1% of the patients with a HA-BSI (Table A5). Among patients with a CA-/HCA-BSI or a HA-BSI, 16.1% and 32.8%, respectively, of the last PA specimens were retrieved more than 2 days before discharge. Still, the median difference between the last PA date and the discharge date was 0 and 1 days, respectively. Sixthly, our results apply to Danish patients only, and may differ in other countries, settings, and healthcare systems. However, the study elucidates patho-physiological mechanisms, which may apply to all humans regardless of different external conditions. Finally, the validity of diagnostic data from the Danish National Patient Registry differs [22], but we believe this is of minor importance in the computation of the CCI as a rough indicator of the patients’ “real” comorbidity status.

## 5. Conclusions

The last PA specimen retrieved during a BSI admission was a strong prognostic predictor for both short- and long-term mortality among patients discharged alive. Little was gained from assessing changes between the first and the last PA specimens. However, a shorter time span between these (i.e., higher velocity of the change in the PA levels) strengthened the discriminatory ability of the last PA level. The same applied to prognostic models restricted to the younger patients and patients with no recorded comorbidity. Our observational study thus raised some interesting issues, for which more specific mechanisms deserve to be evaluated in prospective studies.

## Figures and Tables

**Table 1:** Baseline characteristics of the study population and the nine patient groups based on their first and last plasma albumin level during the admission with the bloodstream infection.

Variable	First/Last Plasma Albumin Level (g/L)									Total
	<25/<25	<25/25–34	<25/≥35	25–34/<25	25–34/25–34	25–34/≥35	≥35/<25	≥35/25–34	≥35/≥35	
N (%)	267 (2.3) ^1^	328 (2.8)	61 (0.5)	471 (4.1)	3483 (30.1)	897 (7.8)	81 (0.7)	2893 (25.0)	3081 (26.6)	11,562 (100.0)
Age, years	71.6 (63.2–80.4) ^2^	69.2 (58.0–79.0)	61.2 (49.9–69.8)	73.6 (65.0–82.1)	74.2 (64.9–82.8)	70.2 (59.3–78.4)	74.7 (63.0–82.5)	73.7 (63.9–81.6)	69.0 (57.1–78.3)	72.0 (61.7–80.8)
Females	99 (37.1)	144 (43.9)	30 (49.2)	211 (44.7)	1548 (44.4)	379 (42.2)	35 (43.2)	1251 (43.2)	1308 (42.5)	5005 (43.3)
Acquisition										
Community	94 (35.2)	172 (52.4)	27 (44.3)	182 (38.6)	1776 (51.0)	455 (50.7)	33 (40.7)	1584 (54.8)	1828 (59.3)	6151 (53.2)
HCA ^3^	106 (39.7)	83 (25.3)	8 (13.1)	164 (34.8)	1197 (34.4)	251 (28.0)	11 (13.6)	758 (26.2)	793 (25.7)	3371 (29.2)
Hospital	67 (25.1)	73 (22.3)	26 (42.6)	125 (26.5)	510 (14.6)	191 (21.3)	37 (45.7)	551 (19.0)	460 (14.9)	2040 (17.6)
CCI ^4^										
0	32 (12.0)	54 (16.5)	15 (24.6)	62 (13.2)	612 (17.6)	213 (23.7)	15 (18.5)	681 (23.5)	908 (29.5)	2592 (22.4)
1–2	92 (34.5)	128 (39.0)	30 (49.2)	182 (38.6)	1348 (38.7)	344 (38.4)	33 (40.7)	1090 (37.7)	1143 (37.1)	4390 (38.0)
>2	143 (53.6)	146 (44.5)	16 (26.2)	227 (48.2)	1523 (43.7)	340 (37.9)	33 (40.7)	1122 (38.8)	1030 (33.4)	4580 (39.6)
Microorganism										
Gram-negative organisms	126 (47.2)	165 (50.3)	18 (29.5)	230 (48.8)	1870 (53.7)	453 (50.5)	32 (39.5)	1550 (53.6)	1598 (51.9)	6042 (52.3)
Gram-positive organisms	113 (42.3)	122 (37.2)	32 (52.5)	185 (39.3)	1351 (38.8)	372 (41.5)	37 (45.7)	1116 (38.6)	1274 (41.4)	4602 (39.8)
Fungi	6 (2.2)	13 (4.0)	3 (4.9)	10 (2.1)	51 (1.5)	19 (2.1)	6 (7.4)	53 (1.8)	20 (0.6)	181 (1.6)
Poly-microbials	22 (8.2)	28 (8.5)	8 (13.1)	46 (9.8)	211 (6.1)	53 (5.9)	6 (7.4)	174 (6.0)	189 (6.1)	737 (6.4)

^1^ Number (%), unless stated otherwise; ^2^ median (inter-quartile range); ^3^ healthcare-associated; ^4^ Charlson comorbidity index.

**Table 2:** Mortality in the study population and the nine patient groups based on their first and last plasma albumin level during the admission with the bloodstream infection.

PA Level (g/L)		First/Last PA Level (g/L)	No. of Patients (%)	Death After Discharge			
First	Last			1–30 Days	31–90 Days	91–365 Days	>365 Days
<25			654 (5.7)	88 (13.5) ^1^	69 (12.2)	98 (19.7)	193 (48.4)
	<25		818 (7.1)	216 (26.4)	108 (17.9)	109 (22.1)	198 (51.4)
		<25/<25	266 (2.3)	64 (24.1)	38 (18.8)	40 (24.4)	75 (60.5)
		<25/25–34	327 (2.8)	24 (7.3)	28 (9.2)	52 (18.9)	97 (43.5)
		<25/≥35	61 (0.5)	0 (0)	3 (4.9)	6 (10.3)	21 (40.4)
25–34			4840 (42.0)	456 (9.4)	442 (10.1)	672 (17.0)	1493 (45.7)
	25–34		6690 (58.0)	491 (7.3)	548 (8.8)	934 (16.5)	2073 (43.9)
		25–34/<25	471 (4.1)	139 (29.5)	62 (18.7)	57 (21.1)	105 (49.3)
		25–34/25–34	3473 (30.1)	291 (8.4)	340 (10.7)	518 (18.2)	1116 (48.0)
		25–34/≥35	896 (7.8)	26 (2.9)	40 (4.6)	97 (11.7)	272 (37.1)
≥35			6039 (52.4)	243 (4.0)	272 (4.7)	648 (11.7)	1728 (35.4)
	≥35		4025 (34.9)	77 (1.9)	127 (3.2)	375 (9.8)	1143 (33.2)
		≥35/<25	81 (0.7)	16 (19.8)	8 (12.3)	12 (21.1)	18 (40.0)
		≥35/25–34	2890 (25.1)	176 (6.1)	180 (6.6)	364 (14.4)	860 (39.6)
		≥35/≥35	3068 (26.6)	51 (1.7)	84 (2.8)	272 (9.3)	850 (31.9)
Total			11,533 (100) ^2^	787 (6.8)	783 (7.3)	1418 (14.2)	3414 (40.0)

^1^ Number (%—for mortality beyond day 30, of remaining patients); ^2^ <11,562 patients of the study cohort as 29 patients emigrated.

**Table 3:** Adjusted hazard ratios (95% confidence intervals) for 1–30, 31–90, 91–365, and >365-day mortality (in bold if significant) in baseline model ^1^ with the plasma albumin (PA) variables.

PA Variable	Category (g/L)	Adjusted Hazard Ratio (95% Confidence Interval)			
		1–30 Days	31–90 Days	91–365 Days	>365 Days
Change, from first to last PA specimen	<25/<25	**3.06 (2.33–4.02)**	**1.86 (1.32–2.61)**	**1.40 (1.01–1.94)**	**1.47 (1.16–1.87)**
	<25/25–34	1.03 (0.68–1.56)	0.99 (0.67–1.46)	1.22 (0.91–1.62)	0.89 (0.73–1.10)
	<25/≥35	NA ^2^	0.74 (0.23–2.31)	0.79 (0.35–1.77)	1.19 (0.77–1.83)
	25–34/<25	**3.67 (2.99–4.51)**	**1.78 (1.35–2.33)**	1.20 (0.91–1.58)	1.15 (0.94–1.40)
	25–34/25–34	Ref.	Ref.	Ref.	Ref.
	25–34/≥35	**0.40 (0.27–0.60)**	**0.48 (0.35–0.67)**	**0.68 (0.55–0.85)**	**0.78 (0.68–0.89)**
	≥35/<25	**2.54 (1.53–4.21)**	1.21 (0.60–2.46)	1.24 (0.70–2.21)	0.86 (0.54–1.38)
	≥35/25–34	**0.79 (0.65–0.95)**	**0.67 (0.56–0.81)**	**0.84 (0.74–0.96)**	**0.80 (0.73–0.87)**
	≥35/≥35	**0.25 (0.19–0.34)**	**0.33 (0.26–0.42)**	**0.60 (0.51–0.69)**	**0.73 (0.66–0.79)**
First PA specimen	<25	**1.59 (1.26–2.00)**	**1.36 (1.05–1.76)**	**1.31 (1.06–1.62)**	1.14 (0.98–1.32)
	25–34	Ref.	Ref.	Ref.	Ref.
	≥35	**0.49 (0.42–0.57)**	**0.53 (0.46–0.62)**	**0.76 (0.68–0.85)**	**0.79 (0.74–0.85)**
Last PA specimen	<25	**3.69 (3.14–4.34)**	**2.02 (1.64–2.49)**	**1.35 (1.11–1.65)**	**1.35 (1.16–1.56)**
	25–34	Ref.	Ref.	Ref.	Ref.
	≥35	**0.31 (0.24–0.40)**	**0.43 (0.35–0.52)**	**0.66 (0.58–0.74)**	**0.83 (0.77–0.89)**

^1^ Model with sex, age, acquisition of the bloodstream infection (community-acquired, healthcare-associated, hospital-acquired), Charlson comorbidity index (0, 1–2, >2 points), microorganism group (*Escherichia coli*, *Enterobacter* spp., *Klebsiella* spp., other Enterobacteriaceae, *Pseudomonas aeruginosa*, anaerobic Gram-negative rods, other Gram-negatives, *Staphylococcus aureus*, coagulase-negative staphylococci, *Streptococcus pneumoniae*, hemolytic streptococci, enterococci, other Gram-positive cocci, Gram-positive rods, Fungi, poly-microbial). ^2^ Not applicable (no deaths in the group).

## Data Availability

The data that support the findings of this study are not openly available due to reasons of sensitivity (GDPR rules). The data are located in controlled-access data storage at Statistics Denmark.

## Abbreviations

The following abbreviations are used in this manuscript:

AUROC	area under the receiver operating characteristic curve
BC	blood culture
BSI	bloodstream infection
CA	community acquisition/acquired
CCI	Charlson comorbidity index
CI	confidence interval
CRP	C-reactive protein
HA	hospital acquisition/acquired
HCA	healthcare association/associated
HR	Hazard ratio
NA	Not applicable
PA	Plasma albumin
RSD	Region of Southern Denmark
SIRS	Systemic Inflammatory Response Syndrome

## Appendix A

**Table A1:** Fluctuations in plasma albumin (PA) levels between the earliest and latest PA specimens for the 6831 patients who did not change between the PA level category groups.

PA Specimen Level		Fluctuation Between Earliest and Latest PA Specimen Level		
Earliest	Latest	Decline	Unchanged	Increase
<25 g/L	<25 g/L	113 (42.3)	44 (16.5)	110 (41.2)
25–34 g/L	25–34 g/L	1894 (54.4)	430 (12.4)	1159 (33.3)
≥35 g/L	≥35 g/L	1972 (64.0)	330 (10.7)	779 (25.3)
Total		3979 (58.3)	804 (11.8)	2048 (30.0)

**Table A2:** Areas under the receiver operating characteristic curve (AUROCs) for 1–30, 31–90, 91–365, and ≥365-day mortality as outcomes.

Model	AUROC (95% Confidence Interval)			
	1–30 Days	31–90 Days	91–365 Days	>365 Days
Baseline ^1^	0.72 (0.70–0.73)	0.73 (0.71–0.74)	0.72 (0.71–0.73)	0.72 (0.71–0.73)
Baseline and first plasma albumin group	0.74 (0.73–0.76)	0.75 (0.73–0.76)	0.72 (0.71–0.74)	0.72 (0.72–0.73)
Baseline and last plasma albumin group	0.78 (0.77–0.80)	0.76 (0.74–0.76)	0.73 (0.72–0.74)	0.72 (0.72–0.73)
Baseline and change between first and last group	0.79 (0.77–0.80)	0.76 (0.75–0.78)	0.73 (0.72–0.74)	0.73 (0.72–0.73)

^1^ Model with sex, age, acquisition of the bloodstream infection (community-acquired, healthcare-associated, hospital-acquired), Charlson comorbidity index (0, 1–2, >2 points), microorganism group (*Escherichia coli*, *Enterobacter* spp., *Klebsiella* spp., other Enterobacteriaceae, *Pseudomonas aeruginosa*, anaerobic Gram-negative rods, other Gram-negatives, *Staphylococcus aureus*, coagulase-negative staphylococci, *Streptococcus pneumoniae*, hemolytic streptococci, enterococci, other Gram-positive cocci, Gram-positive rods, fungi, poly-microbial).

**Table A3:** Area under the receiver operating characteristic curve (95% confidence interval [CI]) for patient subgroups, in bold if the CI overlaps 0.80 or its lower bound is >0.80.

Subgroup	Model	Day 1–30	Day 31–90	Day 91–365	Day >365
Age group					
15–64 years	Baseline ^1^	**0.81 (0.77–0.84)**	**0.82 (0.79–0.85)**	**0.79 (0.77–0.82)**	0.75 (0.74–0.77)
	Baseline & change ^2^	**0.85 (0.82–0.88)**	**0.85 (0.82–0.88)**	**0.81 (0.78–0.83)**	0.77 (0.75–0.79)
65–79 years	Baseline	0.70 (0.67–0.72)	0.72 (0.69–0.74)	0.71 (0.70–0.73)	0.67 (0.65–0.68)
	Baseline & change	**0.79 (0.76–0.81)**	0.75 (0.73–0.78)	0.72 (0.70–0.74)	0.68 (0.66–0.69)
≥80 years	Baseline	0.66 (0.64–0.69)	0.68 (0.65–0.70)	0.64 (0.61–0.66)	0.62 (0.61–0.64)
	Baseline & change	0.74 (0.71–0.76)	0.71 (0.68–0.73)	0.66 (0.64–0.68)	0.63 (0.61–0.64)
Charlson ^3^					
0 points	Baseline	**0.84 (0.80–0.89)**	**0.86 (0.82–0.90)**	**0.79 (0.74–0.83)**	**0.79 (0.77–0.81)**
	Baseline & change	**0.87 (0.83–0.91)**	**0.90 (0.86–0.93)**	**0.79 (0.75–0.84)**	**0.80 (0.78–0.82)**
1–2 points	Baseline	0.71 (0.68–0.74)	0.72 (0.69–0.75)	0.66 (0.64–0.69)	0.68 (0.66–0.70)
	Baseline & change	**0.79 (0.77–0.82)**	0.76 (0.73–0.79)	0.68 (0.65–0.70)	0.69 (0.67–0.70)
≥3 points	Baseline	0.61 (0.59–0.63)	0.63 (0.60–0.65)	0.61 (0.59–0.63)	0.61 (0.60–0.63)
	Baseline & change	0.72 (0.69–0.74)	0.67 (0.65–0.69)	0.62 (0.61–0.64)	0.62 (0.60–0.63)
Quartile ^4^					
First (1–4)	Baseline	**0.79 (0.76–0.82)**	**0.81 (0.78–0.83)**	**0.78 (0.76–0.80)**	0.77 (0.76–0.79)
	Baseline & change	**0.84 (0.81–0.87)**	**0.83 (0.81–0.86)**	**0.79 (0.77–0.81)**	0.78 (0.76–0.79)
Second (5–7)	Baseline	0.73 (0.70–0.77)	**0.78 (0.75–0.81)**	0.74 (0.71–0.76)	0.74 (0.72–0.76)
	Baseline & change	**0.80 (0.77–0.84)**	**0.81 (0.78–0.83)**	0.76 (0.73–0.78)	0.74 (0.72–0.76)
Third (8–16)	Baseline	0.70 (0.67–0.73)	0.71 (0.69–0.74)	0.70 (0.68–0.73)	0.71 (0.69–0.72)
	Baseline & change	**0.77 (0.75–0.80)**	0.75 (0.72–0.77)	0.72 (0.70–0.74)	0.71 (0.70–0.73)
Fourth(17–387)	Baseline	0.71 (0.68–0.74)	0.69 (0.66–0.73)	0.69 (0.66–0.71)	0.68 (0.66–0.69)
	Baseline & change	**0.77 (0.75–0.80)**	0.73 (0.70–0.76)	0.69 (0.67–0.72)	0.68 (0.66–0.70)
Liver ^5^	Baseline	**0.75 (0.69–0.81)**	**0.73 (0.66–0.80)**	0.69 (0.63–0.75)	0.61 (0.57–0.65)
	Baseline & change	**0.82 (0.77–0.88)**	**0.76 (0.69–0.82)**	0.70 (0.64–0.76)	0.66 (0.61–0.70)
Kidney ^6^	Baseline	0.72 (0.67–0.76)	0.69 (0.64–0.74)	0.67 (0.63–0.70)	0.69 (0.66–0.71)
	Baseline & change	**0.78 (0.74–0.82)**	0.71 (0.66–0.75)	0.68 (0.65–0.72)	0.69 (0.67–0.72)

^1^ Model with sex, age, acquisition of the bloodstream infection (community-acquired, healthcare-associated, hospital-acquired), Charlson comorbidity index (0, 1–2, >2 points), microorganism group (*Escherichia coli*, *Enterobacter* spp., *Klebsiella* spp., other Enterobacteriaceae, *Pseudomonas aeruginosa*, anaerobic Gram-negative rods, other Gram-negatives, *Staphylococcus aureus*, coagulase-negative staphylococci, *Streptococcus pneumoniae*, hemolytic streptococci, enterococci, other Gram-positive cocci, Gram-positive rods, fungi, poly-microbial); ^2^ Baseline model and change between the first and last plasma albumin specimen; ^3^ Charlson comorbidity index; ^4^ Quartile (range in days) of time between first and last plasma albumin specimen; ^5^ Patients with liver diseases diagnosed prior to or during the admission with the bloodstream infection; ^6^ Patients with kidney diseases diagnosed prior to or during the admission with the bloodstream infection.

**Table A4:** Baseline characteristics of patients with 0 or 1 plasma albumin specimen retrieved during the admission with their first-time bloodstream infection episode (n = 6060).

Variable	n (%) ^1^
Age, years	68.0 (56.8–77.5) ^2^
Females	2721 (44.9)
Acquisition	
Community	3321 (54.8)
Healthcare-associated	1691 (27.9)
Hospital	1048 (17.3)
Charlson comorbidity index	
0	1627 (26.9)
1–2	2363 (39.0)
>2	2070 (34.2)
Microorganism	
Gram-negatives	3.042 (50.2)
Gram-positives	2569 (42.4)
Fungi	83 (1.4)
Poly-microbial	366 (6.0)
Mortality ^3^	
Day 1–30	171 (2.8)
Day 31–90	190 (3.1)
Day 91–365	420 (6.9)
>Day 365	2386 (39.4)

^1^ Unless stated otherwise; ^2^ median (inter-quartile range); ^3^ based on 6045 patients as 15 emigrated after their discharge.

**Table A5:** Time periods in relation to retrieval of the first and last plasma albumin (PA) specimens.

Text	Community-Acquired and Healthcare-Associated (n = 9522)	Hospital-Acquired (n = 2040)
Days between admission and first PA specimen		
Median (inter-quartile range)	0 (0 0)	0 (0 1)
0	8506 (89.3) ^1^	1437 (70.4)
1	709 (7.5)	200 (9.8)
2	141 (1.5)	99 (4.9)
>2	166 (1.7)	304 (14.9)
Days between last PA specimen and discharge		
Median (inter-quartile range)	0 (0 1)	1 (0 4)
0	5302 (55.7)	737 (36.1)
1	2001 (21.0)	425 (20.8)
2	683 (7.2)	210 (10.3)
>2	1536 (16.1)	668 (32.8)
Days between first and last PA specimen, median (inter-quartile range)	7 (4 12)	26 (14 45)
Days between first and last PA specimen (median) vs. length of stay	0.91 (<10^−5^) ^2^	0.82 (<10^−5^)
First PA specimen retrieved on date of the bloodstream infection	7622 (80.0)	29 (1.4)
Last PA specimen retrieved on date of the bloodstream infection	39 (0.4)	21 (1.0)

^1^ n (%), unless stated otherwise; ^2^ Pearson’s correlation coefficient (*p*-value).
